# With the Wounded: The Growing and Propagation of Bulbs

**Published:** 1916-04-29

**Authors:** George Wm. Dick

**Affiliations:** 10 Morton Street, Leith


### THE EDITOR'S LETTER-BOX.

### With the Wounded: The Growing and Propagation of Bulbs.

*To the Editor of* The Hospital.

Sir,—The citizens of Leith are justly proud of their War Hospital at Seafield. Under the capable and wise guidance of Dr. C. W. Macgillivray and his efficient staff of co-workers much good is being zealously performed. The local Press have spoken volumes in praise of the launching out of the hospital and the means about to be taken to make sure that the hospital will not suffer for want of public support and beneficence in arranging of concerts, etc., to brighten and cheer up the valorous wounded lying within its walls.

The subject of providing recuperative work, if I may so put it, for behoof of our wounded sailors and soldiers, will soon take a definite form, and the time seems more than ripe for the uptaking of suitable and congenial work for those brave fellows maimed, many of them, as the result of this colossal war of nations. The question arises, What about the growing and propagation of bulbs as a means to that end? Undoubtedly in the past we have largely been indebted to Holland for the supply of bulbs to our shores. Personally, I think that the climatic and fertilising South of Ireland lends itself splendidly for the growing of such. Competent authorities might, however, differ. Maybe the powers that be at Whitehall will see the wisdom of doing something in that part of the country on the lines I suggest herein. Even in hybridising plant-life our "Tommies" might show themselves adept horticulturists, and prove eye-openers to many in that branch of floriculture. To my mind floriculture might be made the basis of a fine art in the extensive grounds and conservatories of our convalescent hospitals, etc., among the patients thereof, as each incoming and outgoing patient would find much in the closer study of the God of Nature and the immortality of the soul as exemplified in the cultivation of flowers. Our clergy and medical officers of health might do much also to promote such a scheme, as with the love of flowers ingrained into the hearts of our convalescent sick an inspiration would be afforded them to aspire to lofty ideals and to better dwellings, where sunlight and fresh-air would abound, and thus sound the death-knell of slum-life with its vile outlook. A newer faith and realisation among the people would be the result, and the truth of Cowper's beautiful and significant words would ring true as never before in the annals of the people:

"His purposes will ripen fast,
Unfolding every hour;
The bud may have a bitter taste,
But sweet will be the flower."

I am, etc., George Wm. Dick.

10 Morton Street, Leith, April 22, 1916.

### St. George's Hospital.

*To the Editor of* The Hospital.

